# Higher Plasma Fibrinogen Level at Admission Is Associated with Post-Stroke Depression at Discharge

**DOI:** 10.3390/brainsci12081032

**Published:** 2022-08-03

**Authors:** Juehua Zhu, Li Wang, Han Shao, Xiang Tang, Lulu Zhang, Yun Zhou, Yongjun Jiang, Qi Fang, Xiuying Cai

**Affiliations:** 1 Department of Neurology, The First Affiliated Hospital of SooChow University, Suzhou 215006, China; zhujuehua0216@suda.edu.cn (J.Z.); shaohan111259@163.com (H.S.); tangxiang163yx@163.com (X.T.); zll@suda.edu.cn (L.Z.); zhouyun86588069@126.com (Y.Z.); fangqi_008@126.com (Q.F.); 2 Department of Neurology, Zhangjianggang Fifith People’s Hospital, Suzhou 215600, China; wyyx1026@126.com; 3 Department of Neurology, The Second Affiliated Hospital of Guangzhou Medical University, 250 Changgang East Road, Guangzhou 510260, China; jiangyjnju@gmail.com

**Keywords:** post-stroke depression, fibrinogen, gender, inflammation

## Abstract

Background: Post-stroke depression (PSD) is a common complication of stroke, which seriously affects the functional outcome of patients. Systemic low-grade inflammation associated with PSD has been shown to occur at several months to years, however, whether these inflammatory markers predicted PSD at an acute stage of stroke is controversial. Method: A total of 625 patients with acute ischemic stroke (219 female, 35.40%) were included in this study. PSD was diagnosed using the 17-item Hamilton depression scale (HAMD) at 7 days following discharge (7–14 days after stroke onset). Multivariable logistic regression analysis was applied to build a prediction model for PSD at discharge. Discrimination and calibration of the model were assessed by C-index, calibration plot. Internal validation was conducted using bootstrapping validation. Results: At discharge of hospitalization, 95 patients (15.20%) were diagnosed with PSD. Multivariable logistic regression suggested that female gender (OR = 2.043, 95% CI = 1.287–3.245, *p* = 0.002), baseline NIHSS (OR = 1.108, 95% CI = 1.055–1.165, *p* < 0.001) and fibrinogen (OR = 1.388, 95% CI = 1.129–1.706, *p* = 0.002) were independent predictors for PSD at discharge. The cut-off of the fibrinogen plasma level was 3.08 g/L. These predictors were included in the nomogram. The model displayed good discrimination, with a C-index of 0.730 (95% CI = 0.683–0.777) and good calibration. Conclusion: Female gender, baseline stroke severity and a higher level of fibrinogen were independently associated with PSD at discharge. A nomogram based on these three predictors can be used to provide an individual, visual prediction of the risk probability of PSD.

## 1. Introduction

Post-stroke depression (PSD) is a frequent chronic and recurrent problem that starts shortly after stroke and affect patients’ long-term outcome [1]. The pooled prevalence of PSD is 29–31% at any time within 5 years following stroke [2,3]. Previous literature has revealed that PSD predicts higher mortality, poorer recovery, and lower quality of life for patients, than those without depression [1]. 

Factors influencing the occurrence of PSD are complicated, including pre-stroke factors, such as history of psychiatric illness, female gender; stroke-related factors such as stroke severity, or cognitive impairment; as well as post-stroke factors such as lack of social support [4,5,6]. Research on biological markers of PSD has substantially increased, and the involvement of inflammatory processes has been widely accepted in recent years [6,7].

Laboratory and clinical data have proved that a storm of inflammation develops after ischemic stroke, contributing to secondary brain damage, and leading to a poor prognosis [8,9]. By detecting peripheral blood markers such as cytokines and immune cells, it has been suggested that the immune system plays a role in depressive disorders [10]. 

Systemic low-grade inflammation refers to persistent, chronic inflammatory state, and is a well-known factor for the development of common diseases such as diabetes, atherosclerosis [11], as well as mood disorder [12]. Systemic low-grade inflammation is typically measured via CRP, WBC, coagulation protein fibrinogen, the cytokines interlukin-8 (IL-8), interlukin-6 (IL-6), and tumor necrosis factor α (TNF-α) [11]. 

Evidence demonstrates that elevated inflammatory responses resulting from cerebral ischemia are linked to the pathogenesis of mood disorders [13]. Systemic low-grade inflammation markers such as fibrinogen, white blood cell (WBC) and neutrophil count are significant predictors for 1-year PSD [14]. Additionally, in recent years, peripheral cell counting and their combination, such as neutrophil-to-lymphocyte (NLR), platelet-to-lymphocyte (PLR), or systemic immune–inflammation index (SII), were reported to be able to reflect the inflammatory response, and were associated with the prevalence of PSD [15,16]. 

The factors influencing the development of PSD are diverse, hence there remains a need to develop comprehensive predictor models combining demographic, clinical and biological markers that can better predict PSD and guide early intervention. The frequency of PSD is higher in the first year after stroke and decreases after 12 months. Many studies have focused on the predictors or predicting models for PSD at 3 months or 1 year [14,17]. 

It has not yet been verified whether systemic inflammatory markers are correlated with depression at acute stage post stroke. 

## 2. Materials and Methods

### 2.1. Subjects

All acute ischemic stroke patients admitted in the First Affiliated Hospital of Soochow University from October 2017 to February 2022 were consecutively screened. Six hundred and twenty-five patients were included. Inclusion criteria were: (1) aged between 18 to 80 years old; (2) acute stroke occurred within 7 days of onset, primary or recurrent stroke; (3) stroke diagnosed by brain computed tomography (CT) or magnetic resonance imaging (MRI); and, (4) patients willing to sign the written informed consent and who successfully completed the psychological evaluation. Exclusion criteria included: (1) brain dysfunction caused by other non-vascular causes, such as primary brain tumors, subdural hematoma, paralysis after seizures, metastatic encephaloma, brain trauma, etc.; (2) history of depression and/or other psychiatric illness and dementia, ascertained by research psychiatrists using the Mini-International Neuropsychiatric Interview (MINI); (3) aphasia or severe cognitive dysfunction (if a Mini-Mental State Examination score (MMSE) was ≤19 points); (4) patients diagnosed with transient ischemic attack; and, (5) patients with other concomitant neuropsychiatric disease, such as Parkinson’s disease and epilepsy. 

### 2.2. Clinical Measurement

Demographic variables (age, gender, marriage state, education level); medical information (history of previous depression, previous anxiety, hypertension, diabetes mellitus, dyslipidemia, atrial fibrillation (AF), prior stroke, coronary heart disease, smoking, drinking), and neurological and neuropsychological evaluation (the National Institute of Health Stroke Score (NIHSS) on admission, stroke subtype classified according to TOAST criteria [18]) were collected. Education level was measured by asking participants to report their highest attained educational qualification, which was classified into three levels, namely, primary school or below (no education or primary school), middle school (junior middle school, and/or senior middle school), and college and above (junior college, undergraduate, master’s degree or doctor’s degree). Smoking or drinking was defined as smoking or drinking habits before current stroke onset.

### 2.3. Measurement of Systemic Low-Grade Inflammation Markers

Biological samples were drawn from peripheral venous blood the morning after admission to the stroke unit. The values (WBC in 10^12^/L, lymphocyte in 10^12^/L, neutrophil in 10^12^/L, platelet in 10^12^/L, CRP in mg/L, fibrinogen in g/L, as well as NLR, dNLR, PRL, SII) were collected, allowing us to explore the hypothesis of an association between chronic inflammation state at the acute phase of stroke and PSD. The formulas were as follows: NLR was calculated as neutrophil count/lymphocyte count, dNLR was calculated as neutrophil count/(WBC-neutrophil count), PRL was calculated as platelet count/lymphocyte count, and SII was calculated as platelet count × neutrophil count/lymphocyte count.

### 2.4. Psychological Measurement

All patients were evaluated by the 17-item HAMD [19] before discharge (day 7–14 after the onset of stroke) by two qualified and trained doctors. Patients with a HAMD score over 7 were diagnosed with PSD according to the *Structured Clinical Interview for the Diagnostic and Statistical Manual of Mental Disorders*, 4th edition. Once patients were diagnosed with PSD, they were treated with psychological counseling and/or medication such as selective serotonin (5-hydroxytriptaimine, 5-HT) re-uptake inhibitors (SSRIs) based on their symptoms and severity. Follow-up and re-evaluation were carried out in the outpatient clinic once a month.

### 2.5. Statistical Analysis

Continuous variables are presented as median with interquartile range (IQR), and categorical variables are shown in percentages. Baseline variables on demographic and clinical data were compared by Mann–Whitney *U*-test for continuous variables, and chi-square test for categorical variables. Univariable logistic regression analysis was performed to find variables that accounted for PSD. To adjust for confounding factors with *p* < 0.05, multivariable logistic regression analysis was used to assess any independent factors of PSD. A receiver operating characteristic (ROC) curve was used to test the overall discriminative ability of fibrinogen at admission, for PSD at discharge. Youden index was used to determine an optimal cut-off for fibrinogen on the ROC curve. Statistical analysis was performed using SPSS for Windows, version 22.0 (SPSS Inc., Chicago, IL, USA). Two-tailed significance values were applied, and statistical significance was defined as *p* < 0.05. 

The nomogram was constructed by R version 3.6.0. The nomogram was created by assigning a graphic preliminary score to each of the predictors with a point ranging from 0 to 100, which was then summed to generate a total score, finally converted to the logit and then to an individual probability (from 0 to 100%) of PSD. The performance of the nomogram was evaluated by Harrell’s concordance index (C-index) and the calibration plot [20]. A C-index > 0.7 generally reflected a well-fitted feature of the predictive model. The calibration curve was performed with 1000 bootstrapped resamples for internal validation and was used to analyze the agreement between nomogram and actual observation. The packages of RMS and Hmisc were involved in this process.

## 3. Results

### 3.1. Sample Characteristics

**Figure 1:**
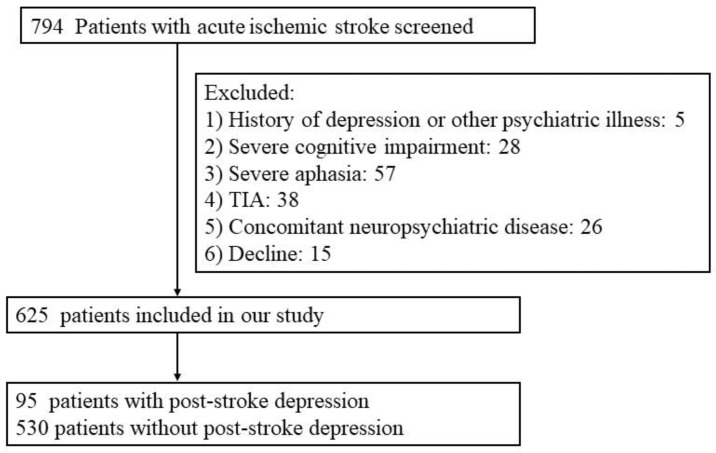
Flow chart. Flow diagram of included and excluded patients.

In the current study, we included 625 ischemic stroke patients (Figure 1), comprising 406 males (64.96%) and 219 females (35.04%). The median (IQR) age was 65 (57–72) years. At discharge of hospitalization, the median (IQR) 17-item Hamilton depression scale (HAMD) score was 1 (0–5) and the incidence of PSD for acute ischemic stroke at discharge was 15.20% (95/625). The baseline characteristics of the 95 patients with PSD at discharge and 530 patients without PSD, are summarized in Table 1. The patients in the PSD group were predominantly female (49.47% vs. 32.45%, *p* = 0.002). The stroke symptoms were more severe in the PSD group (NIHSS, 4 (2–9) vs. 2 (1–4), *p* < 0.001). The level of systemic low-grade inflammation markers fibrinogen (3.21 (2.47–3.67) g/L vs. 2.64 (2.23–3.28) g/L, *p* < 0.001) and CRP 3.69 (1.11–9.03) mg/L vs. 2.24 (0.96–5.66) mg/L, *p* = 0.012) were significantly elevated in the patients with PSD than those without PSD. No significant differences were found for other systemic inflammation markers including white blood cell (WBC) count, lymphocyte count, neutrophil count, platelet count, neutrophil-to-lymphocyte ratio (NRL), derived NRL (dNRL), monocyte-to-lymphocyte ratio (MRL), platelet-to-lymphocyte ratio (PRL) or systemic immune–inflammation index (SII) between PSD and non-PSD groups.

### 3.2. Predictive of the Occurrence of Depression at Discharge

**Figure 2:**
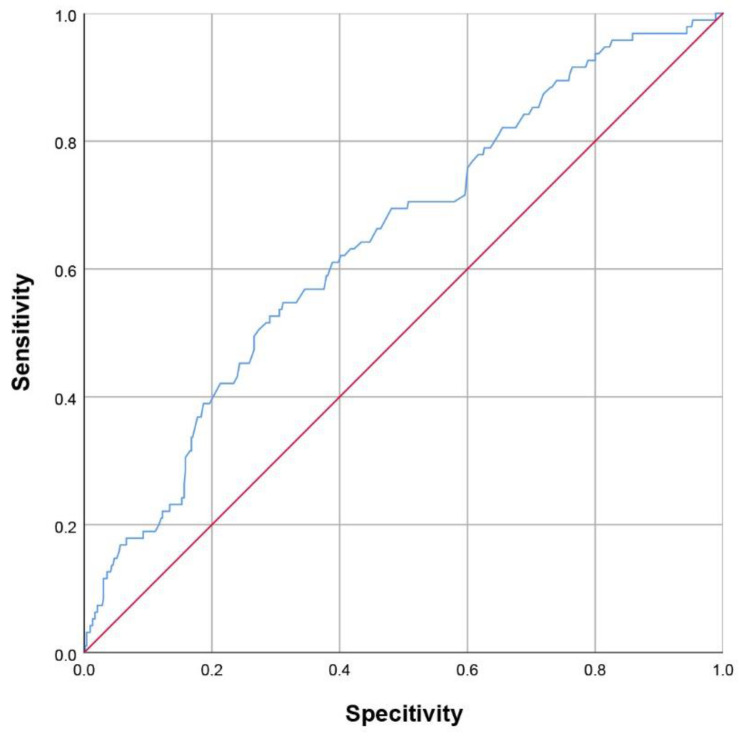
Receiver operating characteristic (ROC) curve analysis for the plasma level of fibrinogen on admission in predicting Post-stroke depression at discharge.

After adjusting for covariates, female gender (OR_adjusted_ = 2.043, 95% CI = 1.287–3.245, *p* = 0.002), baseline NIHSS (OR_adjusted_ = 1.108, 95% CI 1.055–1.165, *p* < 0.001) and level of plasma fibrinogen (OR_adjusted_ = 1.388, 95% CI = 1.129–1.706, *p* = 0.002) could independently predict depression following discharge after ischemic stroke (Table 2). CRP lost statistical importance when adjusted with the above three factors (OR_unadjusted_ = 1.057, 95% CI = 1.020–1.094, *p* = 0.002; OR_adjusted_ = 1.013, 95% CI = 0.969–1.059, *p* = 0.566). The ROC for fibrinogen level at admission demonstrated that the area under the ROC (AUROC) was 0.643 (95% CI = 0.584–0.703), and showed that the optimal cut-off value of fibrinogen was 3.08 g/L (Figure 2) for predicting PSD at discharge (54.7% sensitivity, 68.9% specificity).

### 3.3. Establishment of a Nomogram for PSD at Discharge 

**Figure 3:**
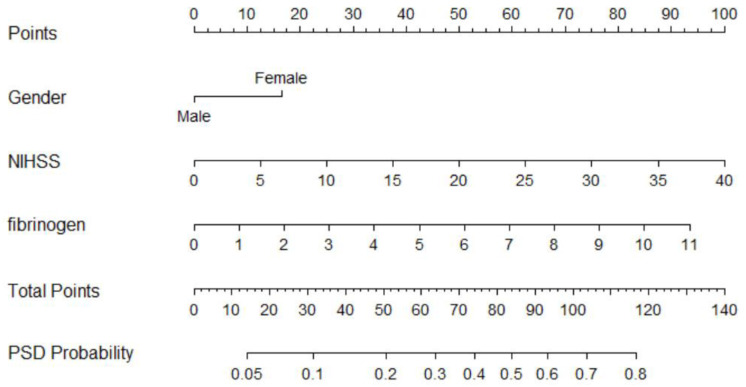
Nomogram predicting Post-stroke depression of acute ischemic stroke at discharge. NIHSS: National Institutes of Health Stroke Scale.

The above significant predictors for PSD at discharge were used to construct the nomogram (Figure 3). The nomogram was generated by assigning a graphic preliminary score to each of the three predictors with a point ranging from 0 to 100%. The individual points were then summed to generate the total score, which was converted into the probability of PSD at discharge (from 0 to 100%). To use the nomogram, first the patient’s value for each predictive variable was identified, then a straight line was drawn upwards from each predictive value to the top point reference line, and the points from each predictor were summed. The location of the sum on the total points reference line was found, and a straight line was drawn from the total points line down to the bottom probability line, to obtain the patient’s probability of PSD.

**Figure 4:**
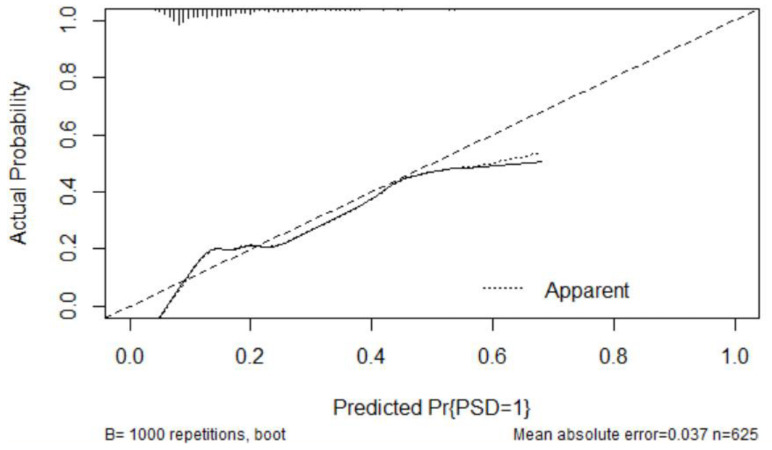
The calibration curve for predicting Post-stroke depression at discharge. NIHSS: National Institutes of Health Stroke Scale.

The discriminatory ability of the current model was assessed using C-statistics. The nomogram derived from our cohort had a C-index of 0.730 (95% CI = 0.683–0.777). The calibration curve for the probability of PSD at discharge is demonstrated in Figure 4. The x-axis presents the predicted PSD resulted from the nomogram, and the y-axis exhibits the actual PSD. The calibration plot reveals a general fit of the nomogram predicting PSD.

## 4. Discussion

In our study, female gender, stroke severity quantitated as NIHSS, and higher baseline plasma fibrinogen level were independent predictors for PSD at discharge, and a nomogram derived from the above parameters was constructed to calculate the probability of PSD at discharge for individual stroke patients.

Data from the South London Stroke Register suggests that incidence of PSD (HAMD ≥ 7) ranges from 7% to 21% per year in the 15 years after a stroke, and the overall frequency of PSD is around 30–32% [1,3,21]. Most depression starts within a year of stroke, and half the patients recover within a year [21]. Meta-analysis data suggests that 28% (23–33%) of depression occurrences start within 1 month after stroke [3], and the incidence of PSD at an earlier stage (within 2 weeks) has been reported as being between 39.3% and 56.63% [22,23]. In these studies, DSM-IV depressive disorders (CIDI-Auto version 2.1), the Korean version of the Beck depression inventory (K-BDI), were used for depression assessment, instead of HAMD. We assumed that the rate of PSD might be underestimated in the current study, partially due to the difference in statistical method and PSD definition. Patients with impairments of speech, cognition or consciousness were excluded as these dysfunctions hindered them from completing the HAMD screening. 

It has been suggested that female sex is one of the mediators of stress susceptibility and depressive disorders [24,25]. Female sex, personal or family history of psychiatric disorders and higher degree of neuroticism are considered as pre-stroke risk factors for PSD [6]. Our data revealed that female sex is an independent predictor of PSD at discharge (OR = 2.008, 95% CI = 1.262–3.149). A recent retrospective cohort study [26] including 174,901 stroke patients also suggested that females were 20% more likely to develop PSD, and the cumulative risk of depression was consistently elevated for females over 1.5 years of follow-up. Furthermore, previous studies also suggested that the inflammatory profiles differed between gender. Females were more vulnerable to the effects of inflammation, and inflammation was more strongly associated with depressive symptoms in females [24]. In our study, 35.04% of the stroke patients were female, and women might be under-represented. We assume this may partially be due to the disadvantage of a single center study and the sex difference of stroke vulnerability. According to the latest epidemiological survey data, the prevalence of stroke among Chinese men is higher than that of women [27] The age-standardized rates of stroke were 1222.2 per 100,000 in men and 1005.7 per 100,000 in women [28]. A previous study about PSD carried out in China, also reported that 35.7% of included stroke patients were female [15], which was similar to our study.

Consistent with previous studies, the current research suggests that stroke severity is an independent and predominant risk factor for PSD at discharge. Large and multiple strokes are predictive of a higher frequency of PSD [6]. In a meta-analysis, severity of stroke (OR 1.12, 95% CI = 1.08–1.16) and level of handicap (OR 1.52, 95% CI = 1.32–1.75) were proved as risk factors for PSD [29]. A chain mediation model suggested that stroke severity affected ischemic stroke patients’ 1-year depressive symptoms, partially through the mediating role of baseline fibrinogen concentration, baseline neutrophil count, or both [14].

Fibrinogen, a soluble 340-kDaglycoprotein synthesized by the liver, is a plasmatic coagulation factor, and an acute-phase protein and inflammatory marker [30]. Fibrinogen is activated in the coagulation system after vascular diseases, and increased fibrinogen is reported after acute ischemic stroke [31,32]. High levels of plasma fibrinogen are related to hemorrhagic transformation [33], enlargement of the infarction [32], early neurological deterioration [34], and poor functional outcome after thrombolysis [35]. In the long run, higher fibrinogen has been proved be associated with the presence and severity of post-stroke cognitive impairment [36]. Meanwhile, a high level of fibrinogen has been associated with depression 1- or 3-months post-stroke onset [37,38,39]. Our study adds to the evidence that fibrinogen is also associated with depression at an earlier stage post-stroke. The underlying mechanism of how fibrinogen causes PSD remains unclear; some researchers have proposed that fibrinogen might be a mediator between stroke severity and inflammatory response, the latter leading to depressive symptomology [14,37].

In a recent meta-analysis of 13 cohort studies, 3536 participants demonstrated an obvious difference between higher CRP levels and PSD over 1 month post-stroke, but not between higher CRP levels and PSD within 1-month post-stroke [17]. A previous meta-analysis including 18,527 patients revealed a significant association between baseline CRP and subsequent depressive symptoms [40]. Kowalska et al. suggested that a higher CRP level was related to depressive symptoms at day 8, but no depressive symptoms 3 months after stroke [41], while another study found that baseline CRP did not predict depressive symptomatology 6 years later, in a sample of 263 older healthy American adults [42]. The relationship of CRP and PSD has not yet been determined. In our study, which focused on the inflammatory markers and depressive symptom at acute stage of stroke, the CRP level was significantly higher in the PSD group, relative to non-PSD group. However, the CRP level lost its significance when adjusted with other predictors such as female sex, stroke severity and fibrinogen level. The differences in research findings might be due to the differences of timing of PSD diagnosis, the diagnostic criteria for PSD, variability of source, measuring method and timing of CRP, and type of stroke. 

The etiology of PSD is believed to be multi-factorial, related to both biological and psycho-social factors [43]. Accumulating evidence has established that abnormal inflammatory response is closely associated with the occurrence of PSD [15,44]. Pre- and post-stroke inflammatory processes result in cell death, persistent neuroinflammation and deranged neuronal networks in mood-related brain regions [45,46]. Previous studies demonstrated that inflammatory markers were associated with PSD from 1 month up to 6 years after stroke onset [12,15]. Our study adds to the evidence that chronic low-grade inflammatory markers fibrinogen and CRP are related to the early occurrence of depressive symptoms within 2 weeks of stroke onset, and furthermore, plasma fibrinogen level at admission is an independent predictor of PSD at acute stage of stroke.

## 5. Conclusions

Depressive symptoms appear at the acute stage of stroke. Female, severe stroke patients, and those who presented higher fibrinogen levels (above 3.08 g/L) were found to have significantly greater risk of developing PSD. It is of great importance to screen for the occurrence of PSD before discharge, as PSD predicts a poor outcome for stroke survivors. Our nomogram model, based on data collected at admission, provides a practical tool for calculating individual risk of PSD at discharge. For those with PSD or high risk of PSD, early intervention (either non-pharmacological or pharmacological) and long-term follow-up, is necessary.

### Limitations

The current study constructed a nomogram to predict PSD at discharge focusing on chronic low-grade inflammation markers. The model was tested using our primary cohort as an internal validation. However, our model remains to be tested using another cohort as an external validation. In addition, females were under-represented in the included population, which might add to the bias of our study, which was a single center study. Moreover, we only evaluated depressive symptoms at discharge. For future study, depression could be measured both at discharge and during long-term follow-up (months to years) after stroke onset, and studied in a longitudinal data. Finally, as inflammatory cascade after acute stroke might affect the level of inflammatory markers as measured in our study, it is worthwhile to further study the relationship between the dynamic changes of inflammatory markers and the long-term evolution of PSD. 

## Figures and Tables

**Table 1:** Baseline characteristics of the study.

	Total (*n* = 625)	Without PSD (*n* = 530)	With PSD (*n* = 95)	*p* Value
**Demographic characteristics**				
Age, year, median (IQR)	65 (57–72)	65 (56–72)	65 (61–70)	0.647
Female sex, *n* (%)	219 (35.04%)	172 (32.45%)	47 (49.47%)	0.002
Marriage, *n* (%)	614 (98.55%)	523 (98.68%)	91 (95.79%)	0.141
Education level				0.632
No education or primary school, *n* (%)	280 (44.80%)	234 (44.15%)	46 (48.42%)	
Middle school, *n* (%)	276 (44.16%)	237 (44.72%)	39 (41.04%)	
College and above, *n* (%)	69 (11.04%)	59 (11.13%)	10 (10.53%)	
**Vascular risk factors**				
Smoking, *n* (%)	175 (28.00%)	156 (29.44%)	19 (20.00%)	0.063
Drinking, *n* (%)	134 (21.44%)	119 (22.45%)	15 (15.79%)	0.174
Hypertension, *n* (%)	452 (72.32%)	382 72.07%)	70 (73.68%)	0.804
Diabetes mellitus, *n* (%)	186 (29.76%)	157 (29.63%)	29 (30.53%)	0.903
Dyslipidemia, *n* (%)	20 (3.20%)	18 (3.40%)	2 (2.10%)	0.753
Atrial fibrillation, *n* (%)	49 (7.84%)	44 (8.30%)	5 (5.26%)	0.408
Previous stroke, *n* (%)	129 (20.64%)	105 (19.81%)	24 (25.26%)	0.270
Coronary heart diseases, *n* (%)	18 (2.88%)	15 (2.83%)	3 (3.15%)	0.745
**Neurological and neuropsychological evaluation**				
NIHSS on admission, median (IQR)	2 (1–5)	2 (1–4)	4 (2–9)	<0.001
TOAST subtype				0.494
LAA	339 (54.24%)	280 (52.83%)	59 (62.10%)	
CE	47 (7.52%)	40 (7.55%)	7 (7.37%)	
SAO	197 (31.52%)	173 (32.64%)	24 (25.26%)	
OC	28 (4.48%)	24 (4.53%)	4 (4.21%)	
SUD	14 2.24%)	13 (2.45%)	1 (1.05%)	
**Laboratory tests**				
WBC (/10^9^/L), median (IQR)	6.98 (5.69–8.48)	6.90 (5.67–8.44)	7.26 (5.79–8.68)	0.143
Lymphcyte (/10^9^/L), median (IQR)	1.64 (1.25–2.08)	1.65 (1.29–2.09)	1.57 (1.18–1.99)	0.384
Neutrophil (/10^9^/L), median (IQR)	4.47 (3.47–5.87)	4.40 (3.43–5.87)	4.70 (3.77–5.78)	0.169
PLT (/10^9^/L), median (IQR)	203 (166–240)	200 (166–240)	208 (164–261)	0.168
Fibrinogen (g/L), median (IQR)	2.70 (2.27–3.37)	2.64 (2.23–3.28)	3.21 (2.47–3.67)	<0.001
CRP (mg/L), median (IQR)	2.36 (0.97–6.17)	2.24 (0.96–5.66)	3.69 (1.11–9.03)	0.012
NRL (%), median (IQR)	2.64 (1.93–3.78)	2.61 (1.92–3.69)	3.02 (2.03–4.43)	0.095
dNRL (%), median (IQR)	1.88 (1.43–2.50)	1.87 (1.43–2.48)	1.97 (1.42–2.77)	0.265
MLR (%), median (IQR)	0.31 (0.22–0.44)	0.31 (0.22–0.44)	0.34 (0.22–0.50)	0.315
SII, median (IQR)	549 (363–818)	534 (359–812)	642 (369–844)	0.097
PRL (%), median (IQR)	124 (96–160)	121 (96–157)	136 (94–175)	0.069

PSD: Post-stroke depression (at discharge, day 7–14 post-stroke onset), TOAST: Trial of Org 10,172 in acute stroke treatment; LAA: large artery atherosclerosis; CE: cardioembolic; SAO: small artery occlusion; OC: other causes; SUD: stroke of undetermined causes; NIHSS: National Institutes of Health Stroke Scale; CRP: C-reactive protein; WBC: white blood cells; PLT: platelet; NRL: neutrophil-to-lymphocyte ratio; dNRL: derived neutrophil-to-lymphocyte ratio; MLR: monocyte-to-lymphocyte ratio; SII: systemic immune–inflammation index; PRL: platelet-to-lymphocyte ratio.

**Table 2:** Univariate and multivariate analysis of clinical characteristics in patients with or without PSD.

PSD	OR_unadjusted_	95% CI	*p* Value	OR_adjusted_	95% CI	*p* Value
Female sex, *n* (%)	2.308	1.311–3.169	0.002	2.043	1.287–3.245	0.002
NIHSS on admission, median (IQR)	1.126	1.072–1.183	<0.001	1.108	1.055–1.165	<0.001
Fibrinogen, median (IQR)	1.462	1.228–1.740	<0.001	1.388	1.129–1.706	0.002
CRP, median (IQR)	1.057	1.020–1.094	0.002	1.013	0.969–1.059	0.566

PSD: Post-stroke depression (at discharge, day 7–14 post-stroke onset), NIHSS: NIH Stroke Scale on admission; CRP: C-reactive protein.

## Abbreviations

PSD	Post-stroke depression
NIHSS	National Institutes of Health Stroke Scale
CI	confidence interval
TOAST	Trial of Org 10,172 in acute stroke treatment
LAA	large artery atherosclerosis
CE	cardioembolic
SAO	small artery occlusion
OC	other causes
SUD	stroke of undetermined causes
NIHSS	National Institutes of Health Stroke Scale
CRP	C-reactive protein
WBC	white blood cell
PLT	platelet
NRL	neutrophil-to-lymphocyte ratio
dNRL	derived neutrophil-to-lymphocyte ratio
MLR	monocyte-to-lymphocyte ratio
SII	systemic immune–inflammation index
PRL	platelet-to-lymphocyte ratio
